# Supplementary material for: Phylogenetic clustering networks among heterosexual migrants with new HIV diagnoses post-migration in Australia
Source: PLoS One. 2020 Sep 1;15(9):e0237469. doi: 10.1371/journal.pone.0237469 (PMC7462279; doi:10.1371/journal.pone.0237469)
Supplement: S1 Table — (DOCX) [file pone.0237469.s001.docx]

**S1 Table. Unadjusted and adjusted odds of phylogenetic clustering among partial Pol sequences in 332 HIV 1 notifications 2005-2014 in heterosexuals with Pol sequences available**

|  | Total  n | Cluster  n (%) | Unadjusted odds ratio  OR (95% CI) p-value | Adjusted odds ratio |
| --- | --- | --- | --- | --- |
| Year of diagnosis |  |  |  |  |
| 2005-2009 | 169 | 60 (36) | 1.0 |  |
| 2010-2014 | 163 | 58 (36) | 1.0 (0.6-1.6) 0.988 |  |
| Evidence of newly acquired infection |  |  |  |  |
| Yes | 45 | 23 (51) | 2.1 (1.1-4.0) 0.021 | 2.2 (1.1-4.4) 0.027 |
| No | 287 | 95 (33) | 1.0 | 1.0 |
| HIV-1 subtype |  |  |  |  |
| B | 125 | 58 (46) | 2.1 (1.3-3.4) 0.001 | 1.9 (1.1-3.5) 0.028 |
| Other | 207 | 60 (29) | 1.0 | 1.0 |
| Region of birth |  |  |  |  |
| Australia/New Zealand | 126 | 52 (41) | 3.1 (1.6-6.3) 0.001 | 1.9 (0.9-4.3) 0.104 |
| South East Asia | 46 | 14 (30) | 2.0 (0.8-4.7) 0.132 | 1.9 (0.8-4.6) 0.146 |
| Sub-Saharan Africa | 71 | 13 (18) | 1.0 | 1.0 |
| Europe/Americas | 36 | 13 (36) | 2.5 (1.0-6.3) 0.046 | 1.7 (0.6-4.6) 0.310 |
| Other | 44 | 22 (44) | 4.5 (1.9-10.4) 0.001 | 4.3 (1.8-10.1) 0.001 |
| Sex |  |  |  |  |
| Male | 188 | 68 (36) | 1.0 |  |
| Female | 144 | 50 (35) | 0.9 (0.6-1.5) 0.785 |  |
| Age (per 10 year increase) | 38 (30-48)^a^ | 38 (30-47)^a^ | 0.9 (0.8-1.1) 0.322 |  |
| CD4 count at diagnosis (per 100 Cell/UL increase) | 268 (86-485)^a^ | 268 (89-533)^a^ | 1.1 (1.0-1.1) 0.276 |  |

^a^median (IQR)
